# Development of a MACE risk prediction model based on CCTA-derived quantitative parameters: a proof-of-concept study

**DOI:** 10.3389/fcvm.2026.1884303

**Published:** 2026-07-21

**Authors:** Tianyang Gao, Mingyu Zou, Wei Zhou, Yu Zhong, Shu Zhou, Sen Xu, Libo Zhang

**Affiliations:** Department of Radiology, General Hospital of Northern Theater Command, Shenyang, China

**Keywords:** coronary artery disease, CTA-derived multiparameters, major adverse cardiovascular events, nomogram, prediction

## Abstract

**Background:**

This study aimed to identify factors influencing major adverse cardiovascular events (MACE) in patients with coronary artery disease (CAD) using quantitative parameters derived from coronary computed tomography angiography (CCTA), and to develop a nomogram-based risk prediction model.

**Methods:**

Clinical data from 280 CAD patients (May 2020–December 2023) were retrospectively analyzed. Based on 1-year follow-up, patients were divided into MACE and non-MACE groups. Baseline characteristics and CCTA-derived parameters were compared, and independent predictors were identified via multivariable logistic regression. A nomogram was constructed and internally validated using Bootstrap resampling, followed by external validation in an independent cohort of 288 patients.

**Results:**

Significant intergroup differences were observed in age, cardiac function grade, smoking history, hypertension history, CT-FFR, stenosis degree, plaque length, total vessel volume, minimal lumen area (MLA), fibrous and fibrofatty plaque volumes, plaque burden (PB), coronary artery calcium score (CACS), perivascular fat attenuation index (FAI), and myocardial mass/volume ratio (MAS) (all *P* < 0.05). Multivariable analysis identified severe stenosis, longer plaque length, larger fibrous plaque volume, and elevated PB, CACS, FAI, and MAS as independent risk factors, while larger MLA was protective (*P* < 0.05). ROC analysis yielded AUCs of 0.735, 0.823, 0.747, 0.704, 0.691, 0.808, 0.810, and 0.689 for stenosis, plaque length, MLA, fibrous volume, PB, CACS, FAI, and MAS, respectively. The combined model achieved an AUC of 0.936, significantly outperforming each single indicator. Internal calibration curves closely matched the ideal line, demonstrating good predictive performance. External validation confirmed robust discrimination (AUC = 0.932) and excellent calibration (Hosmer-Lemeshow *P* = 0.382).

**Conclusions:**

The nomogram based on CCTA-derived quantitative parameters demonstrates high predictive accuracy for MACE in CAD patients and may serve as a reliable clinical screening tool. However, given the short follow-up duration and predominance of soft endpoints (e.g., rehospitalization), these results should be interpreted cautiously regarding hard outcomes, and longer-term prospective studies are warranted.

## Introduction

Among the multiple factors compromising the survival outcomes and long-term prognosis of patients with coronary artery disease (CAD), the incidence of major adverse cardiac events (MACE) is a key clinical priority (1–3). Accordingly, exploring the independent determinants of MACE and constructing robust predictive models are of high clinical significance. With the continuous development of coronary artery imaging technology, research focus has gradually shifted from simple evaluation of coronary artery stenosis to quantitative and qualitative characterization of coronary plaques. Coronary computed tomography angiography (CCTA) allows for clear visualization of coronary plaque features, thus providing credible evidence for the assessment of CAD progression (4–6).

Empirical studies have shown that compared with risk prediction methods based only on clinical risk factors, the prognostic multivariable model that integrates clinical risk factors and quantitative plaque analysis obtained from CCTA has higher accuracy in predicting the prognosis of patients with CAD (7–10). However, few studies have leveraged multiple quantitative CCTA parameters, including stenosis severity, plaque length, fibrous plaque volume, plaque burden (PB), minimum lumen area (MLA), coronary artery calcium score (CACS), perivascular fat attenuation index (FAI), and maximum area stenosis (MAS), concurrently in a single externally validated nomogram. Therefore, the current study aims to construct and externally validate a MACE risk prediction model integrating these CCTA-derived quantitative parameters. Therefore, the current study aims to construct and externally validate a MACE risk prediction model integrating the aforementioned CCTA-derived quantitative parameters. As a preliminary proof-of-concept investigation, we leveraged two existing cohorts (derivation cohort, *n* = 131; external validation cohort, *n* = 288) to explore the feasibility and predictive performance of this multi-parameter integrated model, with the goal of laying the groundwork for future large-scale prospective studies. Nonetheless, this model may provide an initial tool for individualized risk stratification in patients with CAD.

## Materials and methods

### General information

This study adopts a retrospective analysis design. The study cohort consisted of patients who underwent CCTA at our institution between May 2020 and December 2023 with a confirmed diagnosis of CAD. After screening according to pre-established inclusion and exclusion criteria, a total of 280 patients with confirmed CAD were ultimately enrolled in the study. This study protocol was reviewed and approved by the Ethics Committee of the General Hospital of Northern Theater Command (Approval Number: Y[2024]151). Given the retrospective nature of this study, the requirement for written informed consent was waived by the ethics committee; nevertheless, all patients had signed a general informed consent form for the secondary use of their clinical data upon admission.

Inclusion criteria: (1) Patients met the established diagnostic criteria for CAD (11); (2) Patients underwent both invasive coronary angiography (ICA) and CCTA; (3) Complete follow-up data and comprehensive clinical medical records are available for all enrolled patients.

Exclusion criteria: (1) Inadequate image quality of CCTA that precludes reliable diagnostic evaluation; (2) History of coronary artery bypass grafting, pre-intervention or post-intervention status of aortic coarctation, valvular heart disease, anomalous origin of the coronary arteries, and congenital heart disease; (3) Patients with concurrent malignant neoplasms, severe renal dysfunction, or systemic infections during the study treatment period; (4) Inability to comply with scheduled follow-up assessments or absence of complete relevant clinical data.

**Figure 1:**
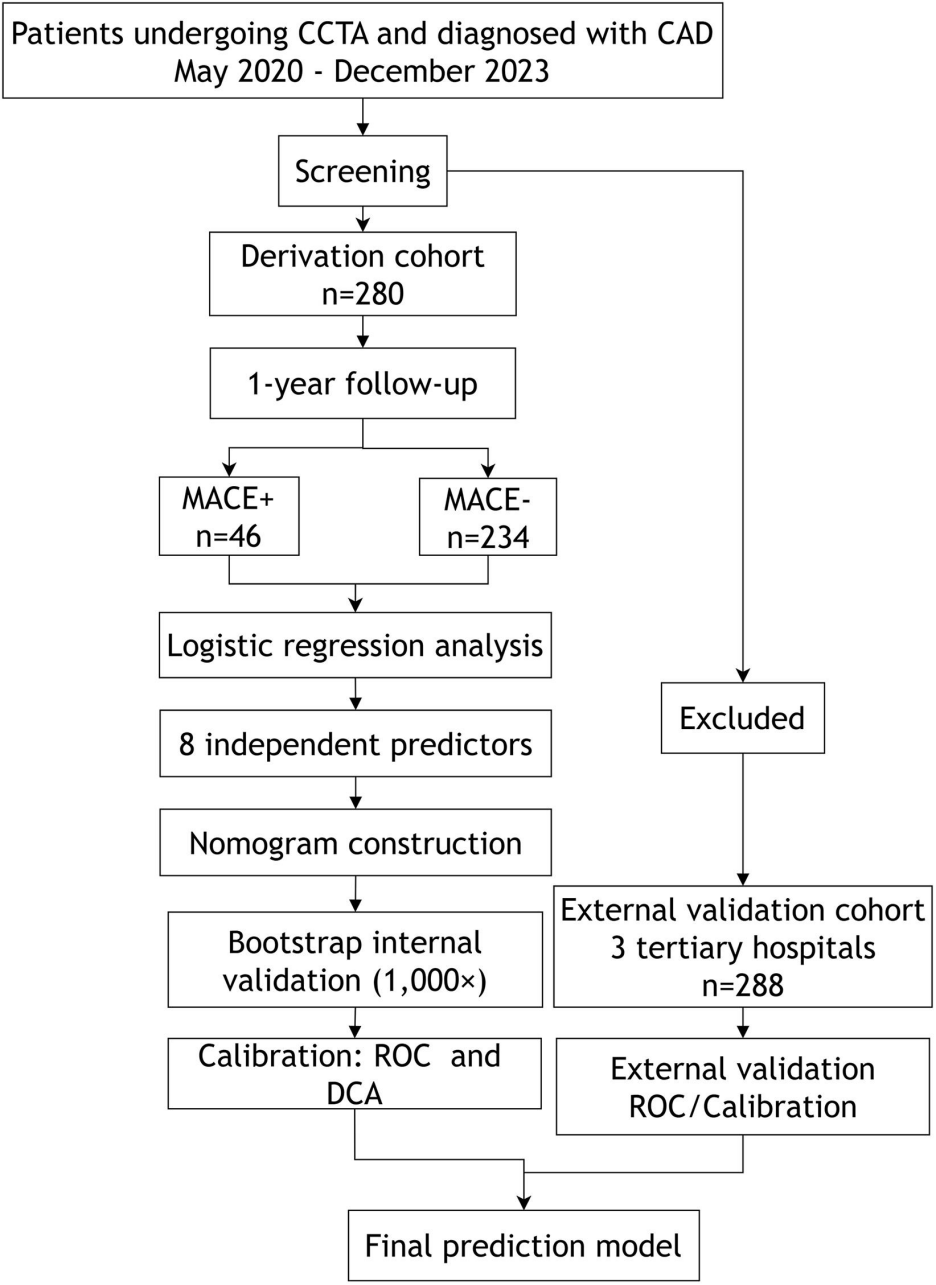
Study flow diagram.

To further verify the generalizability and robustness of the research conclusions presented in this study, we enrolled patients diagnosed with CAD between May 2020 and May 2024 from three external large-scale comprehensive tertiary hospitals, strictly adhering to the same inclusion and exclusion criteria. The three external centers are all tertiary teaching hospitals with experienced cardiovascular imaging departments located in different regions of China. Finally, a total of 288 patients were included in the external validation cohort of this study. A detailed study flow diagram depicting the entire process from patient enrollment to model development and external validation is provided in Figure 1.

### Definition of MACE and post-procedure surveillance protocol

The primary endpoint of this study was the incidence of MACE. MACE was defined as follows: (1) cardiac death including fatal myocardial infarction; (2) non-fatal myocardial infarction; (3) coronary revascularization performed for unstable angina; and (4) rehospitalization attributable to unstable angina (8). Clinical outcomes of all participants were adjudicated by an independent medical committee. In the event of disagreement during outcome assessment, final adjudication was reached via consensus among at least two committee members.

The baseline of follow-up was defined as the date when patients first completed coronary CCTA at our institution. Follow-up was implemented via multiple modalities, including medical record review, consultation with attending clinicians, and telephone interviews with patients and their immediate family members. The follow-up endpoint was defined as either the date of the first occurrence of any outcome event within one year or the pre-specified study termination date (December 31, 2023). During the follow-up period, follow-up assessments were conducted on a monthly basis until the endpoint was reached. Patients who developed MACE within one year were assigned to the positive group, while patients without MACE were allocated to the negative group.

## Radiological image analysis

### Analysis of plaque characteristics

For the derivation cohort, CCTA examinations were performed using a third-generation dual-source CT scanner (Siemens Somatom Force, Siemens Healthineers, Erlangen, Germany). The acquisition parameters were: tube voltage 100–120 kVp, tube current 150–250 mAs, collimation 2 × 192 × 0.6 mm, gantry rotation time 250 ms, and slice thickness 0.75 mm with a reconstruction increment of 0.5 mm. Image reconstruction was performed using a medium-smooth convolution kernel (Bv40) with iterative reconstruction (ADMIRE, strength level 3).

CCTA data were transferred, and multi-planar reconstruction (MPR), curved-planar reconstruction (CPR), volume rendering (VR), and maximum intensity projection (MIP) images were reconstructed. Quantitative and qualitative analyses were conducted on plaques in all diseased coronary arteries identified via CCTA. Plaque analysis was performed using commercially available software (QAngio CT, version 3.0, Medis Medical Imaging Systems BV, Leiden, the Netherlands). All data analyses were performed independently by two senior physicians with extensive clinical experience. All parameters were measured three consecutive times. Quantitative imaging characteristics included the degree of coronary artery stenosis, plaque length, total vessel volume, lumen volume, dense calcified plaque volume, fibrous plaque volume, fibro-lipid plaque volume, MAS, MLA, plaque burden (PB), and CACS (12). Qualitative parameters were defined as follows: (1) low-attenuation plaque; (2) positive remodeling; (3) Napkin-ring sign (NRS, defined as the CT imaging presentation where a low-attenuation plaque is surrounded by a circumferential high-attenuation rim); (4) spotty calcification.

### Measurement of coronary CT-derived fractional flow reserve

CT-FFR analysis was performed using a dedicated computational fluid dynamics-based software platform (cFFR, version 3.0, HeartFlow, Redwood City, CA, USA). The assessment workflow utilizing CT-FFR analytical software initiates with the import of CCTA image data. The software automatically identifies coronary artery centerlines and luminal contours, with manual adjustments accessible when required. A computational algorithm-based model of the entire coronary tree is subsequently constructed, enabling users to obtain the corresponding CT-FFR value by clicking at any target site. Generally, the measurement point should be placed 2–3 cm distal to the distal edge of the plaque, and a CT-FFR≤0.80 is set as the diagnostic cutoff for functional myocardial ischemia (13).

### Measurement of pericoronary fat attenuation index

FAI measurement was performed using an artificial intelligence-based software platform (Syngo.via, version VB30, Siemens Healthineers, Erlangen, Germany). Pericoronary adipose tissue is defined as the adipose tissue located within a spatial boundary equal to the diameter of the adjacent vessel wall, with Hounsfield Unit values ranging from −190HU to −30HU. First, the CCTA data is imported into artificial intelligence software, and the measurement region is realigned to the plaque of interest. Then, the separation distance is adjusted manually, such that the range of pericoronary adipose tissue measurement extends from the vessel wall to a distance matching the vessel diameter. To reduce the potential interference of high-concentration aortic contrast agent on FAI measurement, FAI quantification is performed within a 40 mm long segment starting approximately 10 mm distal to the ostia of three major coronary arteries: the left anterior descending artery, the left circumflex artery, and the right coronary artery.

### External validation cohort imaging consistency

For the external validation cohort, CCTA examinations were performed using CT scanners from three different vendors: Siemens Somatom Force (Siemens Healthineers, Erlangen, Germany), GE Revolution CT (GE Healthcare, Chicago, IL, USA), and Canon Aquilion ONE GENESIS (Canon Medical Systems, Tochigi, Japan). To ensure consistency across centers, all external validation centers used the same image acquisition parameters as the derivation cohort: tube voltage 100–120 kVp, tube current 150–250 mAs, collimation 0.5–0.6 mm, gantry rotation time 250–280 ms, slice thickness 0.5–0.75 mm, and iterative reconstruction algorithms. Plaque analysis, CT-FFR measurement, and FAI quantification in the external validation cohort were performed using the identical software platforms and versions as described above for the derivation cohort.

### Statistical analysis

All statistical analyses were conducted using SPSS 27.0. Categorical data were analyzed with the chi-square test or rank-sum test, and presented as frequencies and constituent ratios. Normality testing for continuous variables was performed using the Shapiro–Wilk test. For normally distributed data, values were expressed as $\left(\bar{x}\pm s\right)$ and compared between groups using the independent samples t-test; for non-normally distributed data, values were expressed as median (interquartile range) [M (P25, P75)] and compared using the rank-sum test. For reproducibility assessment, intraclass correlation coefficients (ICC) were calculated for all quantitative imaging parameters based on 30 randomly selected cases, with both interobserver and intraobserver agreement evaluated. For multivariable analysis, variables with *P* < 0.1 in univariate analysis were entered into the multivariable logistic regression model using the Enter method. Collinearity diagnostics were performed prior to model construction, and all variance inflation factor (VIF) values were <3.5, indicating no significant multicollinearity. A nomogram prediction model was then constructed based on the screened independent risk factors. ROC curve analysis was adopted to evaluate the predictive performance of the nomogram for MACE in CAD patients, and DeLong's test was applied to compare the predictive efficacy between the nomogram model and single parameters. Internal validation of the established prediction model was implemented via the bootstrap resampling method. Calibration curves and decision curve analysis (DCA) were plotted to quantify the performance of the proposed model. For external validation, we additionally plotted the calibration curve and performed the Hosmer-Lemeshow goodness-of-fit test. *P* < 0.05 was set as the threshold for statistical significance.

## Results

### 1-Year incidence of MACE in patients with CAD

Among 280 patients diagnosed with CAD, 46 patients experienced MACE within 1 year of follow-up, corresponding to an incidence rate of 16.43%. The composition of MACE cases is categorized as follows: 1 case of sudden cardiac death (2.17%), 4 cases of non-fatal myocardial infarction (8.70%), 11 cases of unstable angina requiring revascularization (23.91%), and 30 cases of unstable angina requiring rehospitalization (65.22%).

### Comparison of baseline clinical characteristics between the MACE-positive group and MACE-negative group

Comparative analysis of baseline characteristics between the MACE-positive and MACE-negative groups showed no statistically significant intergroup differences in gender distribution, BMI, height, alcohol consumption history, COPD history, diabetes mellitus history, or CAD history (*P* > 0.05). Statistically significant intergroup differences were detected in age, NYHA functional classification, smoking history, and hypertension history (*P* < 0.05). Specifically, the mean age of the MACE-positive group was significantly higher than that of the MACE-negative group (*P* < 0.05). The proportion of patients with NYHA functional class I and II was significantly higher in the MACE-negative group than in the MACE-positive group (*P* < 0.05). Furthermore, the prevalence of smoking history and hypertension history was significantly higher in the MACE-positive group compared with the MACE-negative group (*P* < 0.05). Detailed results are presented in Table 1.

**Table 1:** Comparison of baseline characteristics between the MACE-positive and MACE-negative groups.

Indicators	MACE-Positive (*n* = 46)	MACE-Negative (*n* = 234)	*χ^2^/Z/t*	*P*
Gender (Male/Female)	28/18	163/71	1.369	0.242
Age (year)	58.93 ± 7.20	56.32 ± 6.41	2.29	0.026
BMI (kg/m^2)^	24.87 ± 3.09	23.98 ± 2.90	1.876	0.062
Height(cm)	168.70 ± 5.32	170.24 ± 6.37	−1.742	0.123
NYHA				
I	8 (17.39%）	104 (44.44%)	20.326	<0.001
II	18 (39.13%)	91 (38.89%)		
III	13 (28.26%)	27 (11.54%)		
IV	7 (15.22%)	12 (5.13%)		
Smoking	30 (65.22%)	106 (45.30%)	6.106	0.013
Drinking	27 (58.70%)	134 (57.26%)	0.032	0.858
Hypertension	26 (56.52%)	72 (30.77%)	11.207	0.001
COPD	7 (15.22%)	20 (8.55%)	1.963	0.161
Diabetes	9 (19.57%)	25 (10.68%)	2.842	0.092
Family history of CAD	11 (23.91%)	36 (15.38%)	2.002	0.157

### Comparison of CCTA-derived quantitative parameters between the MACE-positive and MACE-negative cohorts

Comparative analysis of CCTA-derived quantitative parameters between the MACE-positive and MACE-negative cohorts showed no statistically significant differences (*P* > 0.05) in left main coronary artery involvement, lumen volume, dense calcified plaque volume, positive remodeling, low-attenuation plaque, spotty calcification, and the Napkin-ring sign. By contrast, statistically significant intergroup differences (*P* < 0.05) were detected in CT-FFR values, degree of coronary stenosis, plaque length, total vessel volume, MLA, fibrous plaque volume, fibrofatty plaque volume, PB, CACS, FAI, and MAS. The proportion of patients with CT-FFR≤0.80 was significantly higher in the MACE-positive group than in the MACE-negative group (*P* < 0.05). Furthermore, compared with the MACE-negative cohort, the MACE-positive cohort had a significantly higher prevalence of severe coronary stenosis, longer plaque length, larger total vessel volume, greater fibrous and fibrofatty plaque volumes, higher PB, elevated CACS, increased FAI, and augmented MAS (*P* < 0.05), while MLA was significantly lower in the MACE-positive cohort (*P* < 0.05) (Table 2).

**Table 2:** Comparison of quantitative parameters derived from CCTA between the MACE-positive and MACE-negative groups.

Indicators		MACE-Positive (*n* = 46)	MACE-Negative (*n* = 234)	*χ^2^/Z/t*	*P*
CT-FFR	>0.80	10 (21.74%)	110 (47.01%)	10.024	0.002
	≤0.80	36 (78.26%)	124 (52.99%)		
Coronary stenosis	Mild	6 (13.04%)	85 (36.32%)	34.125	<0.001
	Moderate	12 (26.09%)	103 (44.02%)		
	Severe	28 (60.87%)	46 (19.66%)		
Left main coronary artery		21 (45.65%)	106 (45.30%)	0.002	0.965
Plaque length(mm)		22.42 ± 4.67	17.37 ± 3.43	19.557	<0.001
Total vessel volume(mm^3^)		229.45 ± 62.23	194.02 ± 42.26	4.767	<0.001
Lumen volume(mm^3^)		74.00 ± 19.39	72.72 ± 18.19	0.432	0.666
MAS(%)		72.45 ± 7.86	62.48 ± 9.02	6.991	<0.001
MLA(mm^2^)		1.46 ± 0.60	2.13 ± 0.88	−4.939	<0.001
Dense calcified plaque (mm^3^)		8.60 ± 2.07	8.05 ± 1.80	1.847	0.066
Fibrous plaque (mm^3^)		119.79 ± 39.93	95.32 ± 26.47	5.218	<0.001
Fibrofatty plaque (mm^3^)		19.33 ± 5.25	17.87 ± 3.64	2.294	0.023
PB (%)		0.76 ± 0.28	0.63 ± 0.24	3.264	<0.001
CACS (score)		356.21 ± 40.16	316.56 ± 35.47	6.797	<0.001
FAI (HU)		−77.35 ± 6.58	−84.87 ± 6.22	7.425	<0.001
Positive remodeling		19 (41.30%)	68 (29.06%)	2.691	0.101
Low-attenuation plaque		15 (32.61%)	60 (25.64%)	0.952	0.329
Spotty calcification		18 (39.13%)	61 (26.07%)	3.238	0.072
Napkin-ring sign		5 (10.87%)	18 (7.69%)	0.515	0.473

### Reproducibility of imaging measurements

To assess the reproducibility of the imaging measurements, interobserver and intraobserver agreement analyses were performed. For interobserver agreement, all quantitative parameters (including coronary stenosis degree, plaque length, MLA, fibrous plaque volume, PB, CACS, FAI, and MAS) were independently measured by two experienced radiologists (Reader 1 and Reader 2) on 30 randomly selected cases. For intraobserver agreement, Reader 1 repeated the measurements on the same 30 cases two weeks after the initial assessment. The intraclass correlation coefficient (ICC) was calculated for continuous variables, with values interpreted as follows: <0.50, poor; 0.50–0.75, moderate; 0.75–0.90, good; >0.90, excellent. The interobserver ICC values ranged from 0.856 to 0.947, and the intraobserver ICC values ranged from 0.881 to 0.962, indicating good to excellent reproducibility for all measured parameters.

### Univariate analysis of the occurrence of MACE

Discrepant indicators between Tables 1, 2, including age, NYHA functional classification, smoking history, hypertension history, CT-FFR, coronary stenosis degree, plaque length, total vessel volume, fibrous plaque volume, fibrofatty plaque volume, MLA, PB, CACS, FAI and MAS, were individually incorporated into univariate logistic regression models for analysis. Potential predictors of MACE were screened using *P* < 0.1 as the significance threshold. The results indicated that age, smoking history, hypertension history, CT-FFR, coronary stenosis degree, plaque length, total vessel volume, fibrous plaque volume, fibrofatty plaque volume, MLA, PB, CACS, FAI and MAS were all identified as statistically significant predictors (*P* < 0.05; Table 3). To attenuate the impact of multicollinearity in multivariate analysis, the variance inflation factor (VIF) was applied to evaluate collinearity among the 14 independent variables identified via univariate screening. The VIF values of all variables ranged from 1.2 to 3.5 (all <10), confirming the absence of significant multicollinearity, which meets the prerequisite for multivariate regression analysis.

**Table 3:** Univariate analysis of the occurrence of MACE.

Indicators	*B*	*S.E.*	*Wald*	*P*	*OR(*95% CI)
Age	0.060	0.025	5.905	0.015	1.062 (1.012–1.115)
Smoking	0.817	0336	5.960	0.005	2.264 (1.171–4.377)
Hypertension	1.073	0.329	10.615	0.001	2.925 (1.534–5.579)
CT-FFR	−1.332	0.381	12.250	<0.001	0.264 (0.125–0.556)
Coronary stenosis	1.530	0.390	15.414	<0.001	0.217 (0.101–0.465)
Plaque length	0.361	0.056	40.919	<0.001	1.435 (1.285–1.603)
Total vessel volume	0.017	0.004	19.916	<0.001	1.017 (1.010–1.025)
MAS	2.775	0.692	16.998	<0.001	15.235 (4.447–60.369)
MLA	−1.117	0.231	23.315	<0.001	0.327 (0.208–0.515)
Fibrous plaque	0.029	0.006	22.988	<0.001	1.029 (1.017–1.042)
Fibrofatty plaque	0.103	0.040	6.247	0.011	1.108 (1.024–1.200)
PB	2.840	0.689	17.066	<0.001	17.122 (4.439–66.024)
CACS	0.865	0.225	14.780	0.005	2.375 (1.371–3.379)
FAI	1.465	0.545	7.178	<0.001	4.330 (2.712–6.859)

### Multivariable analysis of factors associated with MACE

MACE was defined as the dependent variable, and 14 predictors screened via univariate logistic regression analysis (presented in Table 3)—including age, smoking history, hypertension history, CT-FFR, coronary stenosis degree, plaque length, total vessel volume, fibrous plaque volume, fibrofatty plaque volume, MLA, PB, CACS, FAI, and MAS were designated as independent variables and incorporated into a multivariate logistic regression model. During the data preprocessing stage, we first evaluated the missing value distribution of all included variables. The analysis revealed that the missing rates of continuous variables, namely CT-FFR, MLA, and fibrous plaque volume, were all below 5%, and no missing values were detected in categorical variables (smoking and hypertension history). For missing values in continuous variables, multiple imputation was performed using the Markov Chain Monte Carlo algorithm with 20 iterations. An imputation model containing all variables (including age and coronary stenosis degree) was constructed to retain the correlation structure of the original dataset. Finally, the complete imputed dataset was used for subsequent statistical analysis. The results showed that coronary stenosis degree, plaque length, fibrous plaque volume, PB, CACS, FAI, and MAS were independent risk factors for the occurrence of MACE, while MLA was a protective factor (*P* < 0.05), as detailed in Table 4.

**Table 4:** Multivariate analysis of factors associated With the occurrence of MACE.

Indicators	*B*	*S.E.*	*Wald*	*P*	*OR* (95% CI)
Age	0.052	0.041	1.641	0.200	1.054 (0.973–1.142)
Smoking	0.391	0.552	0.501	0.479	1.478 (0.501–4.356)
Hypertension	1.056	0.576	3.357	0.067	2.875 (0.929–8.896)
CT-FFR	−1.092	0.624	3.064	0.080	0.336 (0.099–1.140)
Coronary stenosis	0.816	0.643	1.613	0.024	0.442 (0.125–1.558)
Plaque length	0.293	0.081	13.098	<0.001	1.341 (1.144–1.572)
Total vessel volume	0.011	0.006	3.252	0.071	1.011 (0.999–1.022)
MAS	1.859	0.536	10.369	<0.001	5.569 (1.258–10.642)
MLA	−1.557	0.432	13.024	<0.001	0.211 (0.090–0.491)
Fibrous plaque	0.031	0.010	10.367	0.001	1.032 (1.012–1.052)
Fibrofatty plaque	0.099	0.064	2.429	0.119	1.104 (0.975–1.251)
PB	2.994	1.100	7.414	0.006	9.967 (2.314–72.318)
CACS	0.941	0.369	6.503	0.025	2.563 (1.273–3.852)
FAI	2.015	0.272	55.236	<0.001	7.501 (1.785–12.348)

### Development of a predictive model for MACE

**Figure 2:**
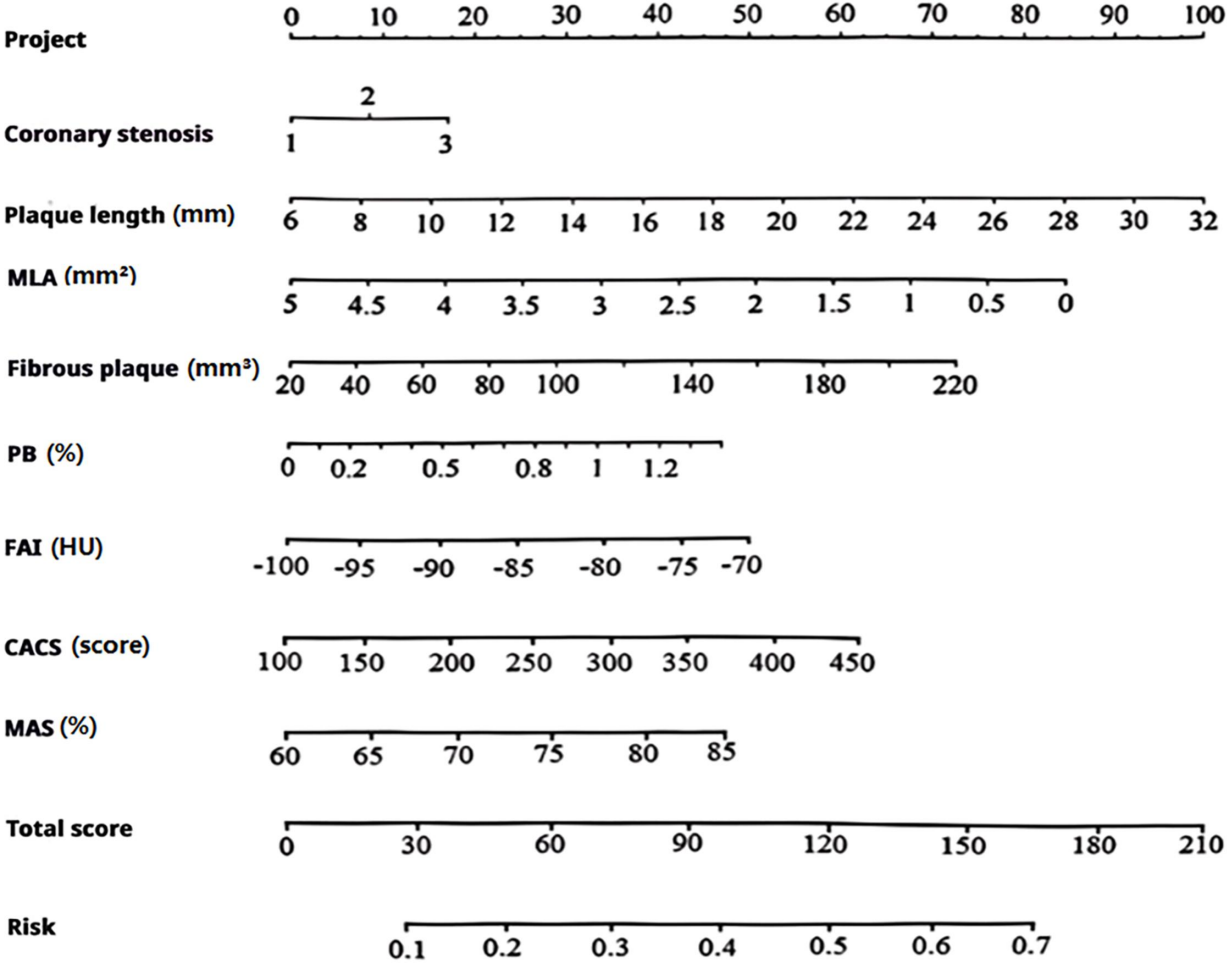
Nomogram for the predictive model of MACE. The scoring system assigns points to each predictor variable based on its value: coronary stenosis (%), plaque length (mm), MLA (mm^2^), fibrous plaque volume (mm^3^), PB (%), FAI (HU), CACS (score), and MAS (%). The total score is calculated by summing the points of all variables, and the corresponding predicted risk is obtained by drawing a vertical line from the total score axis to the risk axis. Risk = 1/(1 + e^−linear predictor^), where the linear predictor is derived from the regression coefficients of the included variables. The risk axis has been extended to 1.0 to accommodate the full range of predicted probabilities.

A predictive model was constructed based on clinical and imaging correlates of MACE, including coronary artery stenosis severity, plaque length, fibrous plaque volume, MLA, PB, CACS, FAI and MAS. Detailed descriptive statistics and visualizations of the above variables are presented in Table 4 and Figure 2, respectively. The linear predictor (LP) is calculated as: LP = −5.124 + (0.816 × coronary stenosis) + (0.293 × plaque length) + (0.031 × fibrous plaque volume) + (2.994 × PB) + (0.941 × CACS) + (2.015 × FAI) + (1.859 × MAS)—(1.557 × MLA), and the predicted risk is obtained as: Risk = 1/(1 + e^(−LP)^). Following Bootstrap internal validation with 1,000 resamples, the optimism-corrected C-index was 0.917, compared with the apparent C-index of 0.936, indicating a modest optimism of 0.019. The calibration slope was 0.924, and the calibration intercept was −0.031, both suggesting good model calibration without significant overfitting.

### Performance analysis and evaluation of predictive models

**Figure 3:**
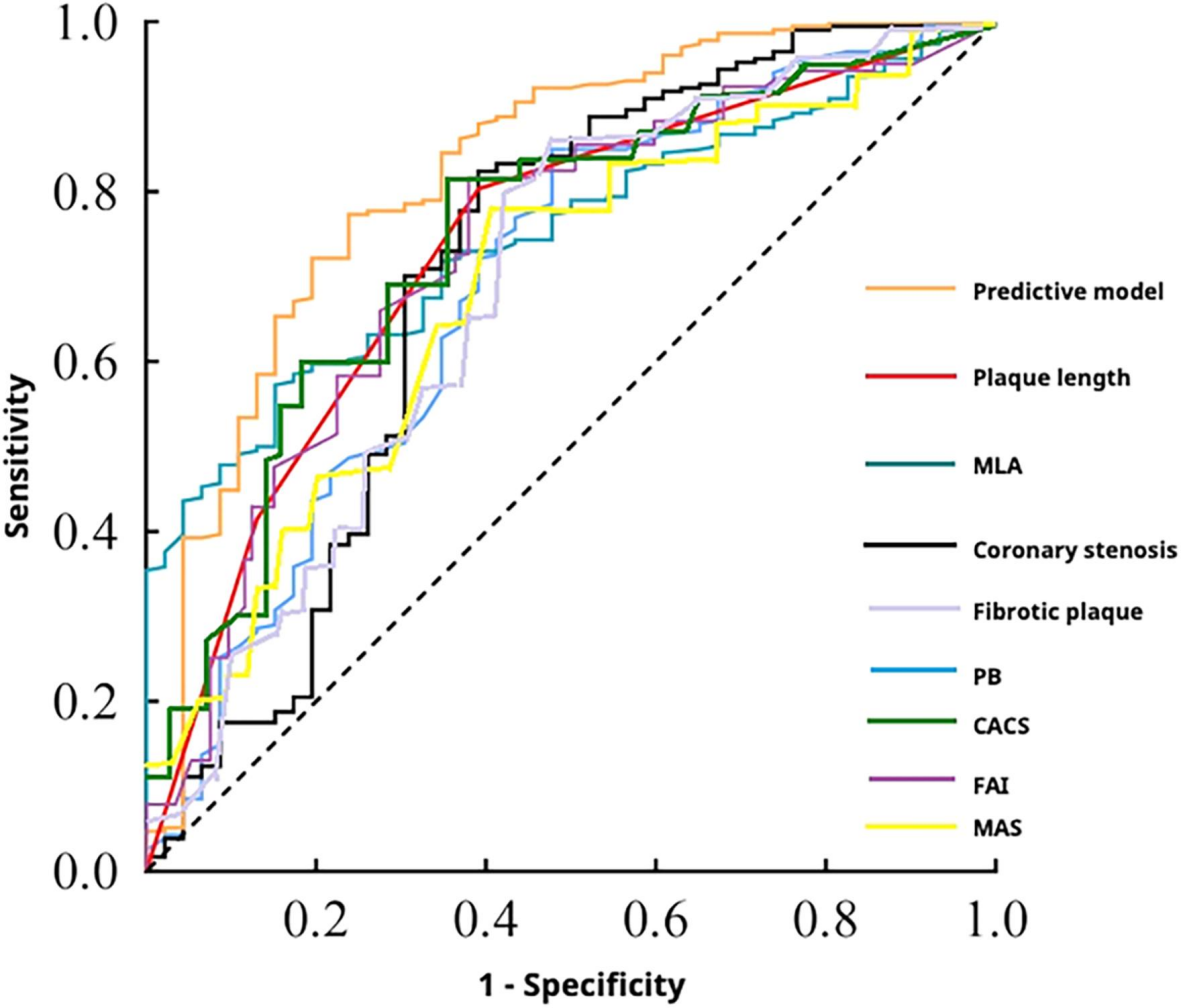
ROC curves of individual predictors and the combined prediction model for MACE in the derivation cohort.

Coronary stenosis severity, plaque length, fibrotic plaque volume, PB, MLA, CACS, FAI, and MAS were adopted as test predictors, and the occurrence of MACE was defined as the outcome variable. ROC curve analysis was performed to assess the predictive performance of each individual candidate predictor and the combined prediction model for MACE occurrence. The results showed that the AUC values for coronary stenosis severity, plaque length, MLA, fibrotic plaque volume, PB, CACS, FAI, MAS, and the combined prediction model were 0.735, 0.823, 0.747, 0.704, 0.691, 0.808, 0.810, 0.689, and 0.936, respectively. The combined prediction model obtained a significantly higher AUC than all individual predictors (*Z* = 2.568, 2.187, 2.446, 2.694, 2.774, 2.205, 2.201, 2.771; *P* = 0.015, 0.024, 0.017, 0.010, 0.008, 0.022, 0.021, 0.006). These results indicate that coronary stenosis severity, plaque length, MLA, fibrotic plaque volume, PB, CACS, FAI, and MAS all have independent predictive value for MACE, and the combined prediction model delivers superior predictive performance (Figure 3). The sensitivity values of coronary stenosis severity, plaque length, MLA, fibrotic plaque volume, PB, CACS, FAI, MAS, and the combined prediction model were 0.609, 0.761, 0.573, 0.609, 0.522, 0.710, 0.725, 0.708, and 0.923, respectively, with the corresponding specificity values of 0.803, 0.774, 0.848, 0.825, 0.850, 0.795, 0.810, 0.835, and 0.804 (Table 5).

**Table 5:** Predictive performance of influencing factors for MACE.

Validation Variables	AUC	*SE*	*P*	95%CI	Youden's index	Sensitivity	Specificity
Coronary stenosis	0.735	0.041	<0.001	0.655–0.815	0.412	0.609	0.803
Plaque length	0.823	0.037	<0.001	0.752–0.895	0.535	0.761	0.774
MLA	0.747	0.033	<0.001	0.683–0.811	0.421	0.573	0.848
Fibrotic plaque	0.704	0.050	<0.001	0.606–0.802	0.434	0.609	0.825
PB	0.691	0.046	<0.001	0.601–0.781	0.372	0.522	0.850
CACS	0.808	0.042	<0.001	0.780–0.879	0.505	0.710	0.795
FAI	0.810	0.040	<0.001	0.783–0.881	0.535	0.725	0.810
MAS	0.689	0.042	<0.001	0.750–0.892	0.543	0.708	0.835
Predictive model	0.936	0.017	<0.001	0.902–0.970	0.727	0.923	0.804

**Figure 4:**
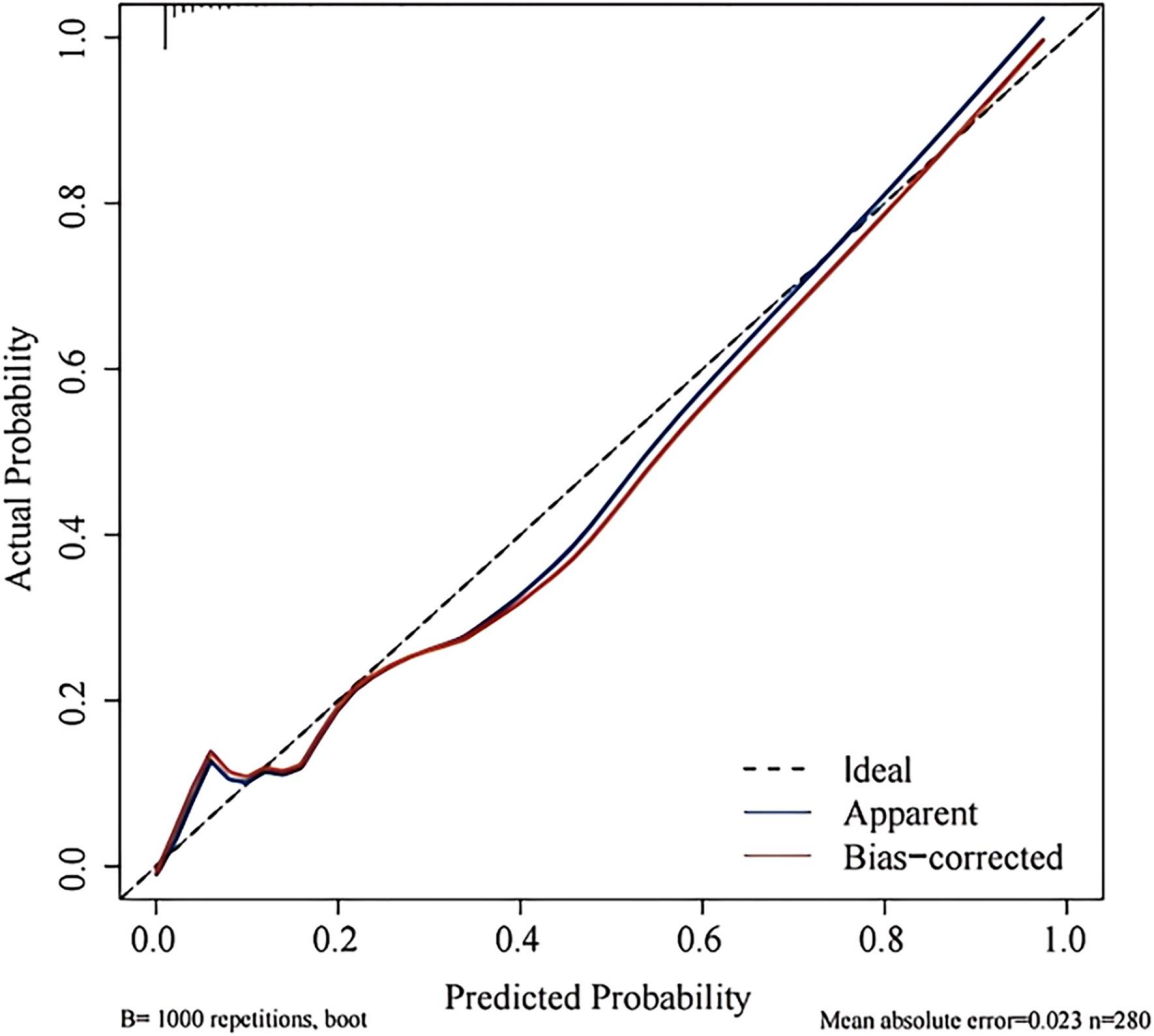
Calibration curve for the prediction model.

**Figure 5:**
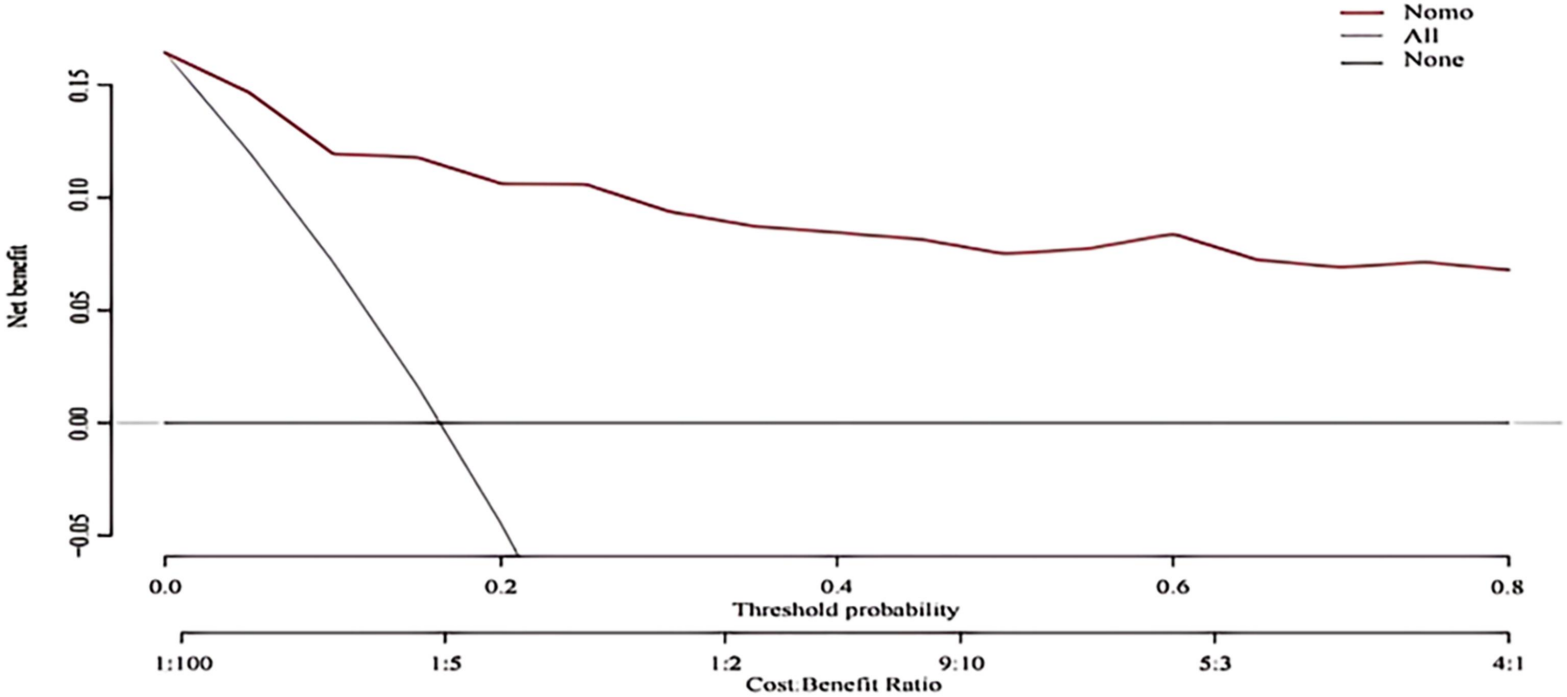
Decision curve analysis for the predictive model.

This study employs the Bootstrap method with 1,000 resamples (*B* = 1,000) to perform internal validation for the constructed prediction model. The calibration curve demonstrates that the predicted probability curve of the model is highly consistent with the ideal reference line, verifying that the model possesses excellent predictive performance (Figure 4). Furthermore, decision curve analysis was conducted to assess the clinical utility of the model, and the results indicate that the model delivers a positive net benefit when the threshold probability ranges from 0.01 to 1.00 (Figure 5).

### External case-based validation

#### 1-Year incidence of MACE in patients with CAD

Among the 288 patients diagnosed with CAD enrolled in the external validation cohort, 91 patients (31.60%) developed MACE. This rate is approximately two-fold higher than that in the derivation cohort (16.43%). The composition of MACE events is specified as follows: 4 cases of sudden cardiac death (4.40%), 11 cases of non-fatal myocardial infarction (12.09%), 10 cases of revascularization for unstable angina (10.99%), and 66 cases of rehospitalization attributable to unstable angina (72.53%).

#### Comparison of baseline clinical characteristics between the MACE-positive group and MACE-negative group

A comparative analysis of baseline characteristics between the MACE-positive and MACE-negative groups revealed no statistically significant intergroup differences in gender, height, BMI, smoking history (*P* > 0.05). In contrast, statistically significant intergroup differences were detected in age, NYHA functional classification, history of COPD, alcohol consumption history, hypertension history, diabetes history and history of CAD (*P* < 0.05). Specifically, the mean age of the MACE-positive group was significantly higher than that of the MACE-negative group (*P* < 0.05). The proportion of patients with NYHA functional class I-II was significantly greater in the MACE-negative group than in the MACE-positive group (*P* < 0.05). Additionally, the prevalence of smoking history, diabetes history and hypertension was significantly higher in the MACE-positive group than in the MACE-negative group (*P* < 0.05). No statistically significant differences were found across the remaining baseline characteristics (Table 6).

**Table 6:** Comparison of baseline characteristics between the MACE-positive and MACE-negative groups.

Indicators	MACE-Positive (*n* = 91)	MACE-Negative (*n* = 197)	*χ^2^/Z/t*	*P*
Gender (Male/Female)	49/42	110/87	1.259	0.264
Age (year)	58.93 ± 7.20	56.32 ± 6.41	2.29	0.026
BMI (kg/m^2)^	24.87 ± 3.09	23.98 ± 2.90	1.876	0.062
Height(cm)	168.70 ± 5.32	170.24 ± 6.37	−1.742	0.123
NYHA				
I	18 (19.78%)	87 (44.16%)	13.326	0.003
II	28 (30.77%)	65 (32.99%)		
III	33 (36.26%)	27 (13.71%)		
IV	12 (13.19%)	18 (9.14%)		
Smoking	60 (65.93%)	106 (53.81%)	3.269	0.071
Drinking	67 (73.63%)	104 (52.79%)	10.355	0.001
Hypertension	66 (72.53%)	75 (38.07%)	28.211	<0.001
COPD	59 (64.84%)	33 (16.75%)	64.006	<0.001
Diabetes	49 (53.85%)	65 (32.99%)	11.316	<0.001
Family history of CAD	31 (34.07%)	29 (14.72%)	14.124	<0.001

#### Comparison of baseline characteristics between the derivation and external validation cohorts

To better understand the substantial difference in MACE event rates between the two cohorts, we compared the baseline clinical characteristics between the derivation cohort (*n* = 280) and the external validation cohort (*n* = 288). As detailed in Supplementary Table S1, the external validation cohort had significantly higher proportions of male patients (*P* = 0.002), smokers (*P* = 0.030), hypertensive patients (*P* = 0.001), patients with COPD (*P* < 0.001), and diabetic patients (*P* < 0.001). No significant intergroup differences were observed in age, BMI, height, drinking history, or family history of CAD (all *P* > 0.05). The distribution of NYHA functional classification did not differ significantly between the two cohorts (*P* = 0.097). These findings indicate that the external validation cohort comprised patients with a greater burden of comorbidities, which may partly explain the higher observed MACE rate in this cohort.

#### Performance and efficacy analysis of predictive models

**Figure 6:**
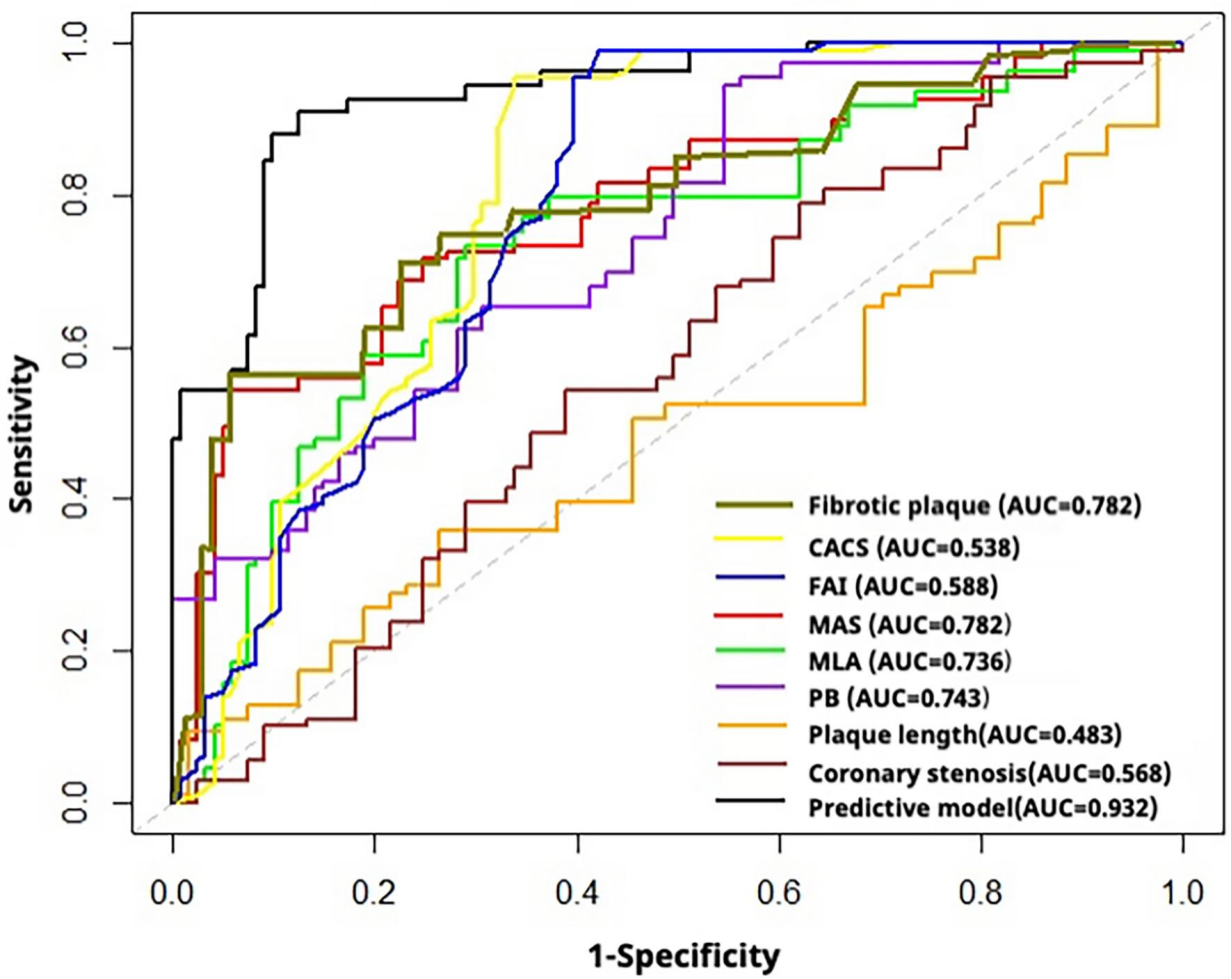
ROC curves of individual predictors and the combined prediction model for MACE in the external validation cohort.

The AUC values for coronary artery stenosis severity, plaque length, MLA, fibrous plaque volume, PB, CACS, FAI, MAS, and the proposed predictive model in this study were 0.568, 0.483, 0.736, 0.782, 0.743, 0.538, 0.588, 0.782, and 0.932, respectively. Notably, the AUC of the predictive model was significantly higher than that of all individual predictors (coronary artery stenosis severity, plaque length, MLA, fibrous plaque volume, PB, CACS, FAI, MAS), with corresponding Z-statistics of 2.578, 3.757, 3.452, 2.454, 2.674, 2.322, 2.454, and 2.634, and adjusted *P*-values of 0.011, 0.014, 0.024, 0.035, 0.042, 0.025, 0.031, and 0.004. These results demonstrate that despite substantial variations in the predictive performance of the aforementioned individual predictors, the integrated model exhibits robust predictive value for MACE, which validates the predictive model constructed in this study (Figure 6).

**Figure 7:**
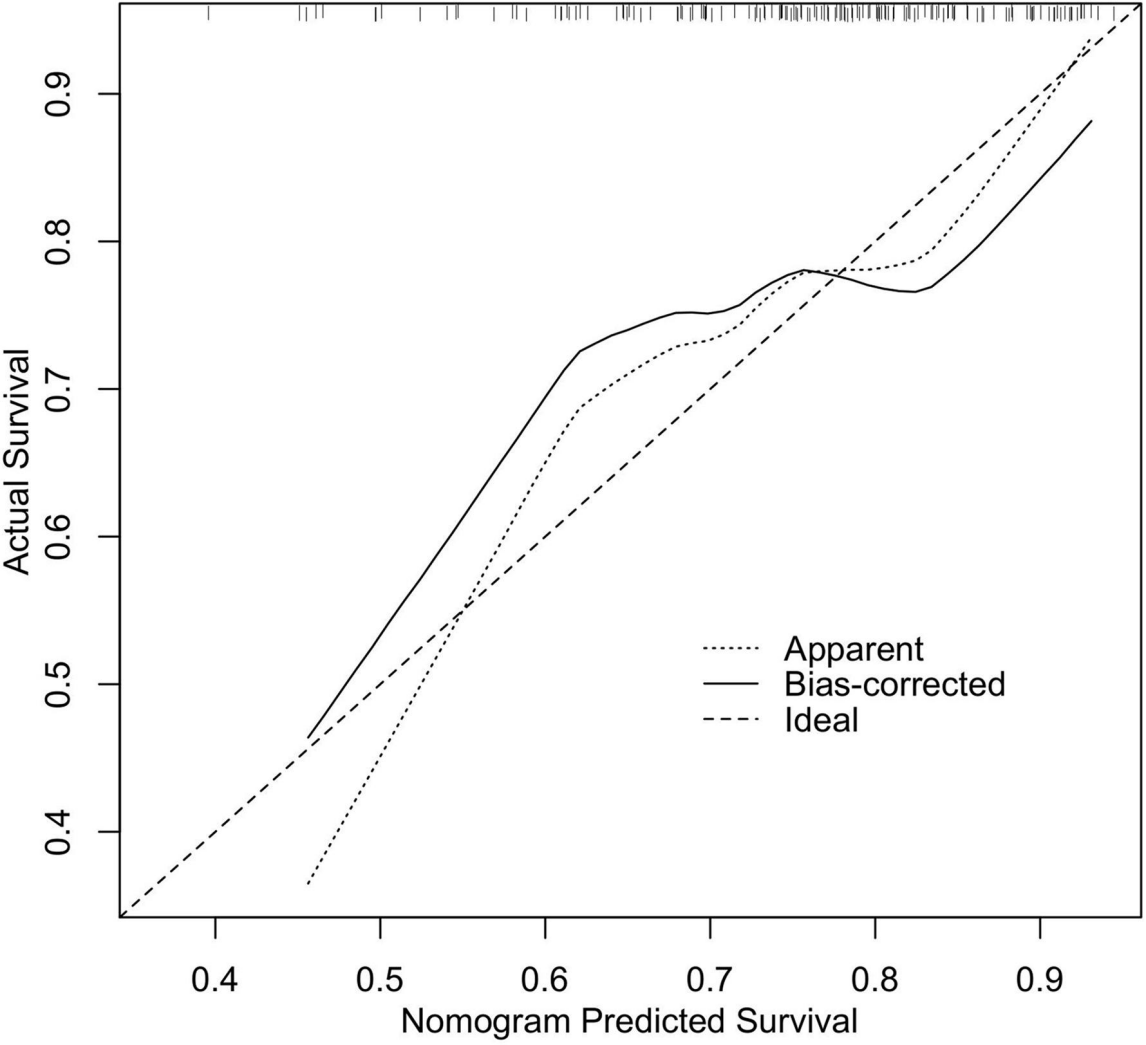
Calibration curve of the prediction model in the external validation cohort.

The sensitivities of the multivariate prediction models for coronary artery stenosis degree, plaque length, MLA, fibrous plaque volume, PB, CACS, FAI and MAS were 0.729, 0.969, 0.519, 0.760, 0.736, 0.930, 0.558, 0.907, and 0.868, respectively. The corresponding specificities were 0.924, 0.152, 0.758, 0.519, 0.554, 0.142, 0.606, 0.232, and 0.979, respectively, as shown in Table 7. The calibration curve for the external validation cohort (Figure 7) demonstrated good agreement between predicted and observed probabilities, with a Hosmer-Lemeshow test *P* value of 0.382, indicating no significant lack of fit.

**Table 7:** Predictive performance of influencing factors for MACE.

Validation Variables	AUC	*SE*	*P*	95% CI	Sensitivity	Specificity	Youden's index
Coronary stenosis	0.568	6.484	0.011	0.468–0.668	0.729	0.924	0.729
Plaque length	0.483	395.765	0.014	0.383–0.583	0.969	0.152	0.121
Fibrotic plaque	0.782	0.072	0.035	0.682–0.882	0.760	0.519	0.279
PB	0.743	0.007	0.042	0.643–0.843	0.736	0.554	0.290
MLA	0.736	0.669	0.024	0.638–0.838	0.519	0.758	0.277
CACS	0.538	0.272	0.025	0.438–0.638	0.930	0.142	0.072
FAI	0.588	0.007	0.031	0.488–0.688	0.558	0.606	0.164
MAS	0.782	0.031	0.004	0.682–0.882	0.907	0.232	0.139
Predictive model	0.932	0.131	0.011	0.832–0.975	0.868	0.979	0.847

## Discussion

Our study is unique in that we integrated eight quantitative CCTA parameters—including stenosis severity, plaque length, fibrous plaque volume, PB, MLA, CACS, FAI, and MAS—into a single nomogram, validated it in an independent external cohort of 288 patients, and achieved a superior AUC of 0.936, suggesting that a multiparametric approach may offer incremental prognostic value over simpler models. To the best of our knowledge, this is the first study to simultaneously incorporate such a comprehensive set of CCTA-derived quantitative parameters into a single externally validated prediction model. This represents a notable advancement over previous reports, most of which have focused on only one or two imaging biomarkers and lacked external validation. Major contemporary CCTA prognostic studies have reported varying AUCs for MACE prediction: the CONFIRM registry demonstrated the incremental value of plaque characterization over stenosis assessment alone (AUC 0.685–0.771) (14), the SCOT-HEART trial showed that CCTA-guided management improved risk stratification (AUC ∼0.80) (15), the PROMISE trial identified high risk plaque features as independent predictors of events (HR ∼2.0–3.0) (16), the PARADIGM registry emphasized the importance of serial plaque progression monitoring (C-statistics 0.668–0.895) (17), and the ICONIC study highlighted the role of high risk plaque characteristics in predicting acute coronary syndromes (18). However, it must be acknowledged that the derivation cohort in this study had a relatively small sample size (*n* = 131) and an events-per-variable (EPV) ratio of approximately 3.3, which to some extent limits the stability of the model. Therefore, the findings of this study should be regarded as proof-of-concept evidence, and their clinical translation warrants caution and further validation in larger-scale studies.

The findings of the current study demonstrate that the severity of coronary artery stenosis is an independent risk factor for MACE. Consistent with this conclusion, Philipp L (19) reported that coronary stenosis with lumen diameter reduction ≥50% acts as an independent predictor of MACE among patients diagnosed with CAD. Progressive coronary stenosis reduces myocardial blood perfusion and aggravates myocardial ischemia, which in turn elevates the risk of MACE (20–22). Myocardial ischemia can induce remodeling of coronary hemodynamics, thereby increasing the likelihood of plaque rupture. Following plaque rupture, released inflammatory mediators and coagulation factors trigger thrombogenesis and vascular occlusion (23, 24). Furthermore, myocardial cell injury induces the secretion of cytokines that promote myocardial fibrosis, which reduces cardiac compliance and consequently increases the risk of arrhythmia and MACE (25).

Increased plaque length induces a reduction in plaque stability, driving the development of unstable plaque phenotypes that can trigger plaque rupture or erosion, which ultimately elevates the risk of MACE (26, 27). Existing studies have confirmed that plaque length is significantly longer in patients with myocardial ischemia than in those without. Longer plaques are correlated with more severe endothelial cell injury at the plaque surface, which promotes the activation of platelets and the coagulation cascade, ultimately facilitating thrombus formation (28, 29). This pathological process can lead to luminal stenosis or complete occlusion, resulting in cardiomyocyte injury or necrosis (30, 31) and further increasing the risk of MACE. Additionally, individuals with longer plaques exhibit significantly more severe cardiac function impairment compared to patients with shorter plaques (32).

A large body of research has confirmed that among patients diagnosed with CAD, individuals with higher fibrotic plaque volumes face an increased risk of MACE within one year post-treatment (33). Fibrotic plaques generally derive from early-stage fatty streaks (34) and progressively enlarge during disease progression. This developmental process can trigger plaque destabilization and rupture, which is followed by thrombus formation, ultimately causing hemodynamic disturbances and worsening myocardial ischemia (35). In addition, the inflammatory response activated during fibrotic plaque formation (36, 37) damages endothelial cell function, resulting in aberrant vasomotor activity. This dysfunction further exacerbates thrombosis and plaque rupture, thereby elevating the risk of MACE (38).

A higher PB is frequently associated with greater vascular stenosis severity and is a well-established independent risk factor for MACE (39, 40). An elevated PB corresponds to an increased total volume of coronary atherosclerotic plaque, which is characterized by expansion of the lipid-rich necrotic core, thinning of the fibrous cap, and heightened plaque vulnerability to rupture (41–43). This process subsequently exacerbates inflammatory responses and activates coagulation cascades, ultimately promoting thrombus formation (44, 45). Furthermore, existing studies confirm that increased PB is correlated with enhanced inflammatory activity, which destabilizes atherosclerotic plaques and triggers rupture, thereby elevating the risk of MACE (46).

MLA is defined as the smallest cross-sectional area of the coronary artery lumen at the site of lesion plaque. This parameter directly reflects the actual effective luminal area available for blood perfusion, and serves as a core indicator for the quantitative assessment of myocardial perfusion. The progression of coronary atherosclerotic plaque causes luminal diameter narrowing, which in turn leads to a reduction in MLA (47, 48). This reduction further decreases blood flow velocity, potentially triggering flow turbulence or even complete flow stagnation (49, 50), and ultimately results in myocardial ischemia and angina pectoris. Furthermore, MLA is closely correlated with plaque stability. Even when the absolute value of MLA is not significantly reduced, increased plaque instability can substantially elevate the risk of MACE in affected patients (51, 52).

The significant correlation between CACS and MACE observed in this study confirms that CACS can serve as a valuable supplementary parameter for cardiovascular outcome prediction. As a core metric for quantifying coronary calcification burden, CACS enables quantitative characterization of calcified plaque volume in the arterial wall via imaging modalities and reflects the cumulative effect of calcium-phosphate metabolic imbalance throughout the progression of coronary atherosclerosis. Conventional perspectives hold that calcified plaques are markers of lesion stability; however, excessive calcification reduces vascular wall compliance and can trigger hemodynamic abnormalities. Simultaneously, stress concentration at the margins of calcified regions elevates the risk of fibrous cap rupture. Particularly for plaques containing non-calcified vulnerable components, the interaction between the “hard” calcified plaque morphology and “soft” non-calcified plaque morphology further increases the probability of plaque rupture and subsequent thrombus formation (53, 54).

A growing body of evidence demonstrates that the epicardial adipose tissue layer acts as a metabolically active endocrine organ, secreting a diverse repertoire of bioactive molecules that mediate inflammatory responses and oxidative stress in the coronary artery wall. The FAI is a quantitative metric for characterizing the CT attenuation of coronary perivascular adipose tissue. FAI values increase as adipocyte lipid content decreases, a process that is accompanied by elevated water content and inflammatory cell infiltration. Accordingly, FAI has been established as a radiological signature of localized inflammatory microenvironments (55). These inflammatory responses are modulated by feedback signaling along the “vessel-adipose tissue” axis, which promotes fibrous cap erosion of atherosclerotic plaques and thereby increases the risk of plaque rupture (56). As a non-invasive quantitative biomarker derived from CCTA, FAI enables the detection of early-stage perivascular inflammation and allows dynamic monitoring of inflammatory regression after clinical intervention (57). In this study, FAI showed a significant correlation with MACE, which supports its potential as a valuable complementary parameter for assessing plaque stability in patients with CAD.

Patients with an elevated MAS present with a significantly higher risk of MACE, a finding consistent with the well-established pathological mechanism of coronary stenosis. Coronary artery stenosis is a multifactorial, dynamically evolving pathological process characterized by accelerated blood flow, turbulent hemodynamics, endothelial dysfunction, and co-activation of inflammatory and coagulation pathways; collectively, these alterations drive the progression of coronary atherosclerosis (58). Elevated MAS values reflect exacerbated myocardial ischemia in myocardial territories perfused by stenotic coronary arteries. Severely stenotic coronary plaques frequently display unstable phenotypes and are predisposed to plaque rupture, which triggers thrombosis and vascular occlusion, ultimately leading to major adverse cardiovascular events. Quantitative assessment of MAS via CCTA has distinct clinical advantages: it enables rapid lesion localization, dynamic monitoring of disease progression and therapeutic response, and yields a more accurate evaluation of the impact of vascular stenosis on myocardial perfusion than conventional stenosis grading systems. This approach provides robust support for clinical decision-making for patients with multivessel coronary artery disease (59).

Regarding CT-FFR, although it was significant in univariable analysis, it did not remain an independent predictor in the multivariable model. This is likely attributable to its strong physiological correlation with stenosis severity and MLA, both of which were retained in the final model. This observation does not diminish the clinical value of CT-FFR, but rather reflects the fact that its prognostic information is largely captured by these anatomical parameters.

### Limitations

The present study has several notable limitations that should be considered when interpreting the findings. First, the modest sample size and the event-per-variable ratio of approximately 3.3 (46 events/14 variables) raise the possibility of overfitting, although Bootstrap internal validation (bias-corrected C-index >0.91) and multiple imputation for missing data suggest that this risk is controlled. None of the eight independent predictors identified in our model showed counterintuitive directions of association, and all are supported by established pathophysiological mechanisms (as detailed in the Discussion section), which further supports the clinical plausibility of our model. Nevertheless, the low EPV ratio remains a limitation. Given the modest sample size, this study should be viewed as a proof-of-concept investigation, and readers should interpret the results with appropriate caution. Second, the one-year follow-up was short, and MACE events were predominantly rehospitalizations for unstable angina (65.2% in derivation, 72.5% in validation) rather than hard endpoints (death or myocardial infarction). Thus, our model is best suited for predicting short-term clinical decision events and requires validation with longer follow-up and harder outcomes. We attempted a sensitivity analysis excluding rehospitalization events, but the model failed to converge due to the limited number of hard endpoint events (*n* = 5 in the derivation cohort). Third, the MACE rate in the external validation cohort (31.6%) was twice that in the derivation cohort (16.4%). As shown in Supplementary Table S1, the external cohort had significantly higher proportions of male patients (*P* = 0.002), smokers (*P* = 0.030), hypertensive patients (*P* = 0.001), patients with COPD (*P* < 0.001), and diabetic patients (*P* < 0.001), reflecting its origin from tertiary referral centers. Encouragingly, the model maintained excellent discrimination (AUC 0.932) and good calibration (Hosmer-Lemeshow *P* = 0.382) in this higher-risk population, supporting its generalizability, though recalibration may be needed when applied to lower-risk outpatient settings. Fourth, the nomogram requires calculation of the linear predictor to convert total score to absolute risk, which limits bedside use; a web-based calculator is being developed to address this. Finally, all patients were Chinese, so validation in other ethnic populations is needed.

## Conclusions

The severity of coronary artery stenosis, lesion length, fibrous plaque volume, PB, CACS, FAI and MAS have been identified as independent risk factors for MACE. In contrast, a larger MLA has been confirmed as a protective factor against MACE. The MACE risk prediction model constructed based on quantitative parameters obtained from CCTA shows stable and reliable predictive performance, which provides a valuable reference for clinical prognostic evaluation and patient risk stratification. Early identification of high-risk patients allows clinicians to implement targeted preventive and therapeutic interventions, and ultimately improves patients' survival outcomes and quality of life.

Future studies should extend the follow-up duration and incorporate more hard endpoints to validate the model's long-term predictive value. Additionally, developing a web-based calculator based on this nomogram will facilitate its clinical application. Furthermore, the applicability of this model in other ethnic populations and lower-risk outpatient settings warrants further validation through multicenter studies.

## Data Availability

The original contributions presented in the study are included in the article/Supplementary Material, further inquiries can be directed to the corresponding author/s.
