# Supplementary material for: Development of a MACE risk prediction model based on CCTA-derived quantitative parameters: a proof-of-concept study
Source: Front Cardiovasc Med. 2026 Jul 21;13:1884303. doi: 10.3389/fcvm.2026.1884303 (PMC13433181; doi:10.3389/fcvm.2026.1884303)
Supplement: Supplementary file 1 [file Table1.docx]

Supplemental Table S1. Comparison of Baseline Characteristics Between the Derivation and External Validation Cohorts

| Indicators |  | Derivation Cohort (n=280) | External Validation Cohort (n=288) | *χ²/Z/t* | *P* |
| --- | --- | --- | --- | --- | --- |
| Gender (Male/Female) |  | 191 / 89 | 159 / 129 | 9.274 | 0.002 |
| Age (years) |  | 56.75 ± 6.54 | 57.14 ± 6.89 | 0.697 | 0.486 |
| BMI (kg/m²) |  | 24.13 ± 2.95 | 24.26 ± 3.01 | 0.523 | 0.601 |
| Height (cm) |  | 169.95 ± 6.25 | 169.08 ± 6.11 | -1.685 | 0.093 |
| **NYHA** | Ⅰ | 112 (40.00%) | 105 (36.46%) | 7.8514 | 0.097 |
|  | Ⅱ | 109 (38.93%) | 93 (32.29%) |  |  |
|  | Ⅲ | 40 (14.29%) | 60 (20.83%) |  |  |
|  | Ⅳ | 19 (6.78%) | 30 (10.42%) |  |  |
| Smoking |  | 136 (48.57%) | 166 (57.64%) | 4.730 | 0.030 |
| Drinking |  | 161 (57.50%) | 171 (59.38%) | 0.205 | 0.651 |
| Hypertension |  | 98 (35.00%) | 141 (48.96%) | 11.278 | 0.001 |
| COPD |  | 27 (9.64%) | 92 (31.94%) | 42.897 | <0.001 |
| Diabetes |  | 34 (12.14%) | 114 (39.58%) | 56.011 | <0.001 |
| Family history of CAD |  | 47 (16.79%) | 60 (20.83%) | 1.503 | 0.220 |
